# Defoliation severity is positively related to soil solution nitrogen availability and negatively related to soil nitrogen concentrations following a multi-year invasive insect irruption

**DOI:** 10.1093/aobpla/plaa059

**Published:** 2020-11-13

**Authors:** Emma Conrad-Rooney, Audrey Barker Plotkin, Valerie J Pasquarella, Joseph Elkinton, Jennifer L Chandler, Jaclyn Hatala Matthes

**Affiliations:** 1 Department of Biological Sciences, Wellesley College, Wellesley, MA, USA; 2 Harvard Forest, Harvard University, Petersham, MA, USA; 3 Department of Earth and Environment, Boston University, Boston, MA, USA; 4 Department of Environmental Conservation, University of Massachusetts Amherst, Amherst, MA, USA

**Keywords:** Defoliation, gypsy moth, insect disturbance, *Lymantria dispar*, nitrogen, soil solution nitrogen

## Abstract

Understanding connections between ecosystem nitrogen (N) cycling and invasive insect defoliation could facilitate the prediction of disturbance impacts across a range of spatial scales. In this study we investigated relationships between ecosystem N cycling and tree defoliation during a recent 2015–18 irruption of invasive gypsy moth caterpillars (*Lymantria dispar*), which can cause tree stress and sometimes mortality following multiple years of defoliation. Nitrogen is a critical nutrient that limits the growth of caterpillars and plants in temperate forests. In this study, we assessed the associations among N concentrations, soil solution N availability and defoliation intensity by *L. dispar* at the scale of individual trees and forest plots. We measured leaf and soil N concentrations and soil solution inorganic N availability among individual red oak trees (*Quercus rubra*) in Amherst, MA and across a network of forest plots in Central Massachusetts. We combined these field data with estimated defoliation severity derived from Landsat imagery to assess relationships between plot-scale defoliation and ecosystem N cycling. We found that trees in soil with lower N concentrations experienced more herbivory than trees in soil with higher N concentrations. Additionally, forest plots with lower N soil were correlated with more severe *L. dispar* defoliation, which matched the tree-level relationship. The amount of inorganic N in soil solution was strongly positively correlated with defoliation intensity and the number of sequential years of defoliation. These results suggested that higher ecosystem N pools might promote the resistance of oak trees to *L. dispar* defoliation and that defoliation severity across multiple years is associated with a linear increase in soil solution inorganic N.

## Teaser

A severe, multi-year gypsy moth population irruption occurred in a temperate New England forest that caused intense leaf herbivory and tree defoliation. We found that trees and forest stands with lower soil nitrogen concentrations experienced more defoliation than those with higher soil nitrogen concentrations. The inorganic nitrogen in soil solution was strongly positively correlated with defoliation intensity and the number of sequential years of defoliation. This could suggest that higher nitrogen pools might promote the resistance of oak trees to defoliation and that defoliation severity across multiple years is associated with a linear increase in soil solution inorganic nitrogen.

## Introduction

Forests are an important global carbon sink that buffer the accumulation of atmospheric carbon dioxide (CO_2_) from the combustion of fossil fuels causing climate change (IPCC 2014). The uptake and sequestration of CO_2_ during the regrowth of Northeastern US forests following agricultural abandonment over the last two centuries continues to play a significant role in reducing the global greenhouse gas burden (Goodale *et al.* 2002; Pugh *et al.* 2019). The carbon sequestered by forest growth is only possible through important interactions with ecosystem nitrogen (N) cycling (Melillo *et al.* 2011). However, the ability of trees to grow and sequester carbon can also be impacted by disturbances such as invasive insect outbreaks, which are potentially increasing in frequency and severity with climate change (Seidl *et al.* 2017).

Gypsy moth (*Lymantria dispar*) is an invasive forest insect that was introduced to New England in the late 1860s, and since then *L. dispar* has periodically exhibited large irruptions during which populations increase by several orders of magnitude (Elkinton and Liebhold 1990; Liebhold *et al.* 2000). During a population irruption, *L. dispar* caterpillars consume large quantities of leaves, and extensive leaf herbivory often leads to widespread defoliation at the spatial scale of entire forest stands (Elkinton and Liebhold 1990). During the summers of 2015–18, forests of Southern New England experienced sustained irruptions of *L. dispar*, where the defoliated forest area was estimated to have at least doubled each year from 2015 to 2017 (Pasquarella *et al.* 2018b). Oaks (*Quercus* spp.) are the preferred hosts for *L. dispar* caterpillars, and although trees can typically withstand a single year of defoliation, successive years of *L. dispar* defoliation can lead to oak tree mortality (Barron and Patterson 2008; Dietze and Matthes 2014; Morin and Liebhold 2016).

Nitrogen (N) is a critical nutrient for both caterpillar and tree growth. Nitrogen from soil organic matter becomes available to tree roots through microbial decomposition that converts organic N into soluble inorganic ammonium (NH_4_^+^) and nitrate (NO_3_^−^) that trees take up from the soil solution through their roots (Bormann *et al.* 1977). Inorganic N taken up by tree roots is used to create the enzyme rubisco and pigments in leaves for photosynthesis. Nitrogen is also a critical nutrient for animals, such as *L. dispar* caterpillars, since they have high N requirements for protein synthesis (Mattson 1980). Therefore, the N concentrations of oak leaves could influence the extent and/or intensity of caterpillar herbivory and potentially play a role in the intensity of overall forest defoliation (Lindroth *et al.* 1997; Foss and Rieske 2003; Milanović *et al.* 2014).

In laboratory environments, *L. dispar* caterpillars sometimes prefer leaves with higher leaf N concentrations, but higher leaf N concentrations can also suppress the relative caterpillar leaf consumption rates. Lindroth *et al.* (1997) found that higher leaf N concentrations suppress relative caterpillar leaf consumption rates (though only for males) and increase caterpillar survival and development rates. Laboratory-feeding preference studies have found that *L. dispar* caterpillars preferentially consume the *Quercus* species with the highest leaf N concentrations for some *Quercus* assemblages (Milanović *et al.* 2014), but other foliar differences might be more important than N concentrations for caterpillar consumption in other *Quercus* species assemblages (Foss and Rieske 2003). While these experiments provide some insight into caterpillar herbivory and N dynamics in a laboratory context, behaviours under field conditions may differ.

In addition to the potential direct effects of leaf N concentrations on rates of herbivory, defoliation by *L. dispar* caterpillars also indirectly influences ecosystem N cycling. Herbivory redistributes N from tree leaves to the soil organic N pool through the production of frass (caterpillar faeces) and the accumulation of insect biomass which can subsequently become available to trees through microbial decomposition and mineralization. Insect herbivory can also either increase or decrease rates of N availability within soils through complex direct and indirect feedbacks (Hartley and Jones 2008).

Overall, higher inorganic soil N availability within a particular site could potentially increase *L. dispar* caterpillar herbivory (and hence internal N cycling rates) through higher leaf N concentrations. Alternatively, *L. dispar* caterpillar herbivory might decrease with higher N availability if caterpillars become satiated more quickly with higher N leaves and reduce their overall consumption as in laboratory studies (Lindroth *et al.* 1997). This study investigated the relationships among *L. dispar* herbivory, defoliation severity, soil and leaf N concentrations, and soil solution inorganic N availability at both individual trees and forest plots in a region defoliated by *L. dispar* in the years 2015–18. We tested two hypotheses assessing the relationships among defoliation and N concentrations and soil solution N at the scale of both individual trees and forest plots:

1. Higher concentrations of leaf and soil N are associated with higher rates of herbivory within trees, and higher concentrations of soil N are associated with higher rates of defoliation at the scale of forest stands.
2. Higher rates of inorganic N availability in soil solution are positively correlated to plot-scale defoliation intensity, since caterpillar herbivory should deliver a pulse of labile N to the forest floor.

To test these hypotheses, we measured the total N concentrations of leaves and soil and soil solution N availability in mixed temperate forests within areas that had been impacted by the *L. dispar* irruption in 2015–18. At individual trees we measured the extent of leaf herbivory, total leaf N concentrations and total soil N concentrations. Within forest plots we measured total soil N concentrations and rates of accumulation of soil solution N, and we assessed plot-level defoliation using a forest condition assessment product based on Landsat imagery (Pasquarella *et al.* 2017).

## Methods

### Study design and measurements of individual trees

Data for this study were collected at two scales: (i) individual red oak (*Quercus rubra*) trees and associated soil in the Amherst, MA, USA area and (ii) oak-dominated mixed forest plots in Central Massachusetts, USA. At each of these scales we measured N concentrations, herbivory and/or defoliation that are detailed in the following sections (Table 1). At the individual tree scale, 12 red oak (*Q. rubra*) trees growing alongside suburban roads and fields in the Amherst, MA area were selected as part of a previous study of *L. dispar* populations and their defoliation of oak trees. In early October 2018, we visually classified the percentage of overall tree defoliation on each tree as the percent of the tree canopy that was missing, presumably due to defoliation. The defoliation had been caused almost entirely by *L. dispar* larvae in May and June 2018. At the same time, leaf samples (*n* = 30) were collected by hand from reachable branches around each tree to gather a spatially distributed representative sample and the percentage of leaf herbivory for each sampled leaf was visually scored by percent categories for herbivory in 10 % increments (e.g. 0–9 % consumed, 10–19 % consumed, etc.) in the laboratory. The average percentage of herbivory among the leaves for each tree was then multiplied by the number of leaves in each herbivory category by the midpoint percentage of the category (e.g. 4.5 % for the 0–9 % category) to estimate the average percent herbivory for each tree.

**Table 1.** Data were collected at individual trees and within forest plots.

Individual trees	Forest plot
• Leaf N concentrations (% N) • Soil N concentrations (% N) • Leaf herbivory (% herbivory) • Tree defoliation (% defoliation)	• Soil N concentrations (% N) • Soil solution inorganic N (mg kg^−1^ day^−1^) • Defoliation intensity (data product from satellite imagery)

### Study design and measurements of trees in forest plots

**Figure 1.**
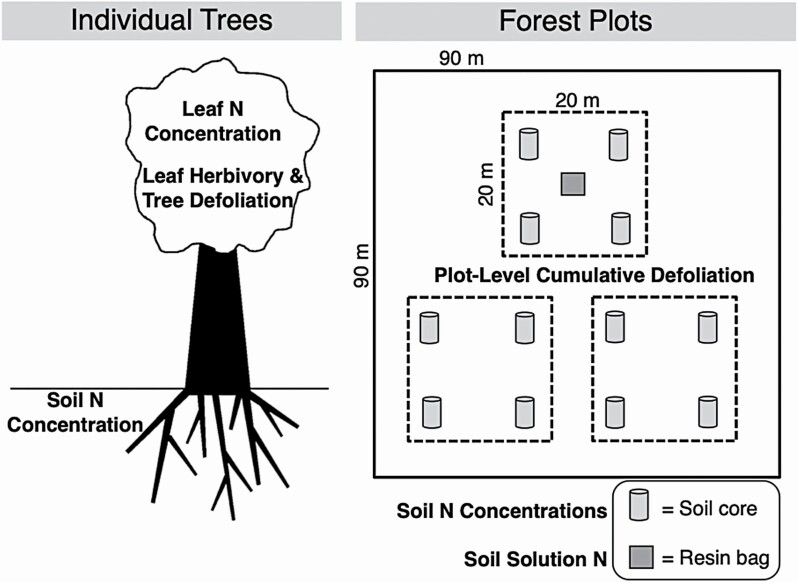
Conceptual diagram outlining the data collected at the scales of individual trees in the Amherst, MA area (*n* = 12) and forest plots in Central Massachusetts (*n* = 9).

At the forest plot scale, ten 90 m × 90 m plots were established in 2019 in Central Massachusetts near the Quabbin Reservoir and the Harvard Forest to study the effects of sequential years of *L. dispar* defoliation. Each 90 m × 90 m plot was composed of three 20 m by 20 m subplots of oak-mixed forest (Fig. 1). One of the 10 plots was excluded from the analysis because it had only two oak trees in it (2.5 % of the trees in the plot were oaks), and therefore the drivers of *L. dispar* defoliation could have been different for this plot compared to the oak-mixed forest plots. All other forest plots had between 12 and 28 oak trees larger than 5 cm in diameter (mean = 20 cm, SD = 6.20 cm) and oaks comprised 15–33 % of the total trees in these plots.

These plots were selected to capture a range of defoliation intensities and durations, informed by data from a forest condition assessment product derived from Landsat imagery (Pasquarella 2017). We used data from this forest condition assessment product to quantify the cumulative severity of growing season defoliation at each plot across the 2015, 2016, 2017 and 2018 growing seasons (Pasquarella 2018a). Specifically, this satellite-based data product averaged the changes in growing season vegetation greenness relative to a fitted baseline model for 2000–10 at each Landsat pixel in the broader New England region (Pasquarella 2017). To facilitate interpretation of results in this analysis, we multiplied the values of the data product by −1 so that a higher value represented a larger disturbance (i.e. as opposed to a more negative number representing a larger negative departure from mean greenness). Thus, the interpretation of the numeric values from this data product are analogous to a statistical *z*-score, where larger values reflect a larger departure from baseline conditions and are associated with more severe defoliation by *L. dispar* (Pasquarella 2017; Pasquarella *et al.* 2018b). For each plot, we calculated the average growing season defoliation for each year (2015–18) as the average of the data product pixels within a 45-m radius of the main plot centre. To represent the cumulative defoliation intensity within each plot, we summed the average growing season departures from mean greenness for the 2015–18 time period (*sensu*Foster 2017).

For some analyses, we used a categorical version of the Landsat-based forest condition assessment product, which separated the continuous greenness departure values into four greenness change classes: slight change (deviations 1–2 model root-mean-squared error; RMSE), moderate change (deviations 2–3 times model RMSE), large change (3–4 times model RMSE) and very large change (4–5 times model RMSE) (Pasquarella 2017, 2018a). For each plot, we calculated the average change class for each year (2015–18) as the average of the data product pixels within a 45-m radius of the main plot centre. We then classified plots as being defoliated within each year if the average change category was ‘moderate change’ or higher. Across the 2015–18 growing seasons, we calculated the total number of years defoliated for each plot as the sum of years that were classified as defoliated. This value of the number of years each plot was defoliated was used in conjunction with the cumulative defoliation intensity metric for some analyses.

### Soil and leaf nitrogen concentrations

To measure soil N concentrations for individual trees and forest plots, we collected samples of the soil organic horizon up to 10 cm depth using a 5-cm-diameter split PVC corer and a mallet. For the Amherst individual trees, three soil cores were taken from around each tree 0.5 m away from the trunk in different directions. If a tree was close to the road (less than about 3 m from road), the side of the tree closest to the road was avoided. The three soil cores associated with each tree were then homogenized into one composite sample. For each of the Quabbin forest subplots (Fig. 1), four soil samples were collected 5 m inward towards the centre of the plot from each of the four plot corners and then homogenized into a composite sample. Soil samples were dried for 24 h at 100 °C, sieved through a 2-mm sieve, and ground and homogenized with a ball mill for 5 min. For each soil sample, a 10-mg subsample was then packed into tins for elemental analysis and measured for the percentage of carbon and nitrogen in each soil sample (vario Micro Cube, Elementar Analysensysteme GmbH).

We also measured the N concentrations of leaves collected in October 2018 that were scored for herbivory from individual Amherst trees. It is important to note that since they were collected at the end of the growing season, they could have started senescing. The 30 leaves from each tree that were scored for herbivory were pooled and ground and homogenized together using a Wiley mill. Next, the ground leaves were more finely ground with a ball mill for 15 min. Then, 2 mg of each leaf sample were packed into elemental analysis tins and analysed for the percent nitrogen content (vario Micro Cube, Elementar Analysensysteme GmbH).

### Soil solution N with resin bags

In order to measure soil solution N over time in the forest plots, we used an ion exchange resin bag method to capture mobile nitrate and ammonium using standard methods (Orwig *et al.* 2008; Templer and McCann 2010). For each resin bag, 10 g of resin were added to a packet created with a mesh stocking. A resin bag was buried 5–10 cm below the soil surface at each of the 10 Quabbin forest plots in the centre of the first subplot in early June (from 11 to 14 June). At each plot, the resin bag was collected 75–78 days (~2 months) after initial burying date to quantify accumulation of soil solution N during a time period that reflects the total soil solution N accumulated during the peak growing season (Sanders-DeMott *et al.* 2018).

After field collection, nitrate (NO_3_^−^) and ammonium (NH_4_^+^) were extracted from the resin using 2 M KCl following Orwig *et al.* (2008). We measured the resulting KCl extracts for NO_3_^−^ and NH_4_^+^ concentrations with an Astoria-Pacific Discrete Analyser using colorimetric analysis (Astoria-Pacific, Inc., Clackamas, OR, USA). Each sample was run twice for replication and quality assurance. The resulting concentrations of NO_3_^−^ and NH_4_^+^ in ppm were converted to mg per kg dry resin, the two replicates were averaged, and that value was divided by the number of days that the resin bag was deployed in order to get the average rates of NO_3_^−^ and NH_4_^+^ production in mg per kg dry resin per day during the June–August summer growing season period. To calculate the total rate of inorganic N production for each resin bag, we added the mean rates of NO_3_^−^ and NH_4_^+^ availability.

### Statistical analyses

We calculated Pearson’s correlation coefficients to test for correlations among N concentrations and soil solution N and defoliation metrics. We fit one-way ANOVA models with *post hoc* Tukey honestly significant differences tests to determine whether the number of years a plot experienced defoliation (1, 2 or 3 years, defined as a categorical variable) explained variation in plot-level soil N concentrations or soil solution N. For soil and leaf % N variables we log-transformed the data before statistical testing. All statistical analyses were conducted and figures were produced in R (R Core Team 2019); we used base R (R Core Team 2019) in addition to R packages dplyr and tidyr to organize (Wickham *et al.* 2019; Wickham and Henry 2020), ggplot2 and cowplot to visualize data relationships (Wickham 2016; Wilke 2019) and agricolae to conduct the Tukey HSD test (de Mendiburu 2019). We defined the alpha value as 0.1 due to our small sample size, and throughout the text we report the actual *P*-value and *r* or *r*^2^ value with each statistical test. We also used packages rgdal and raster in R to calculate the cumulative defoliation scores and to quantify the number of years defoliated from the Landsat-derived data products (Pebesma and Bivand 2005; Bivand *et al.* 2013, 2019; Hijmans 2020).

## Results

### Leaf and soil total N concentrations and defoliation at individual trees

**Figure 2.**
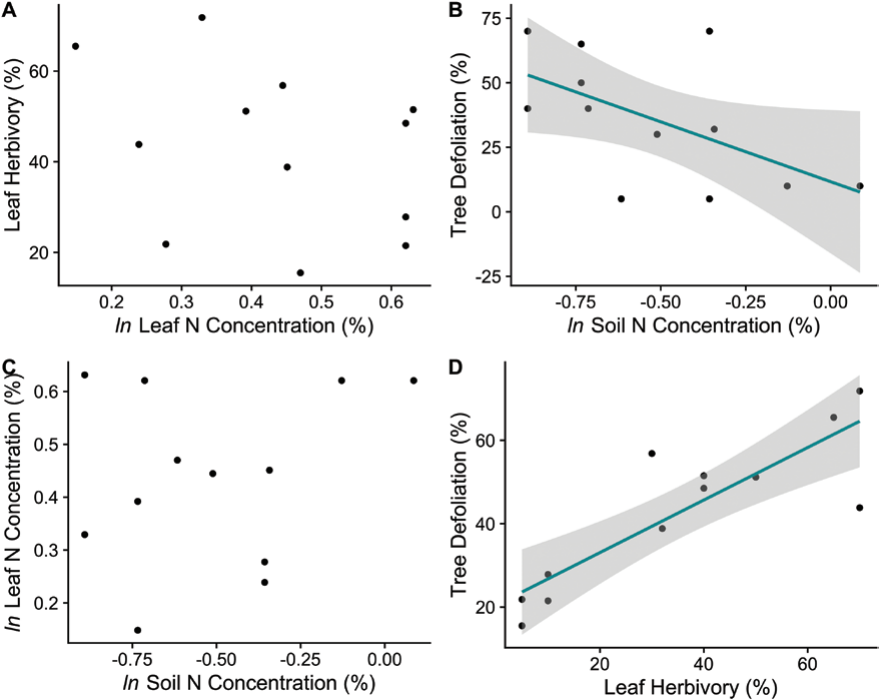
Comparisons among *Q. rubra* leaf herbivory, tree defoliation, leaf % N and soil % N at the individual tree level. Each data point represents an individual tree and the soils associated with them (*n* = 12 trees). (A) Percent herbivory for individual leaves was not associated with leaf % N (*P* = 0.26). (B) The percentage of tree defoliation decreased as the amount of soil % N increased (*P* = 0.052). (C) There was no significant relationship between the leaf % N and soil % N (*P* = 0.47). (D) The measurements of individual leaf herbivory are significantly correlated with defoliation of the entire tree (*P* < 0.01).

Within individual *Q. rubra* trees measured in this study, there was no significant relationship between the extent of leaf herbivory and the N concentrations within leaves (Fig. 2A, *r* = −0.35, *P* = 0.26). This result is contrary to the hypothesis that more herbivory occurs on leaves with higher N concentrations. Tree defoliation had a significant negative correlation with soil total N concentrations, where at higher values of soil total N concentrations there was less total tree defoliation (Fig. 2B, *r* = −0.57, *P* = 0.052).

We found no significant relationship between soil total N concentrations and leaf total N concentrations at individual trees (Fig. 2C, *r* = 0.23, *P* = 0.47). Overall, there was larger variation in the soil total N concentrations (mean = 0.624 %, SD = 0.204, CV = 32.8 %) than the leaf total N concentrations (mean = 1.57 %, SD = 0.255, CV = 16.2 %). We found a statistically significant positive correlation between leaf-level herbivory and overall tree-level defoliation that supported a strong scaling relationship from the leaf to canopy scale (Fig. 2D, correlation = 0.86, *P* < 0.01).

### Soil N cycling and plot-level defoliation

**Figure 3.**
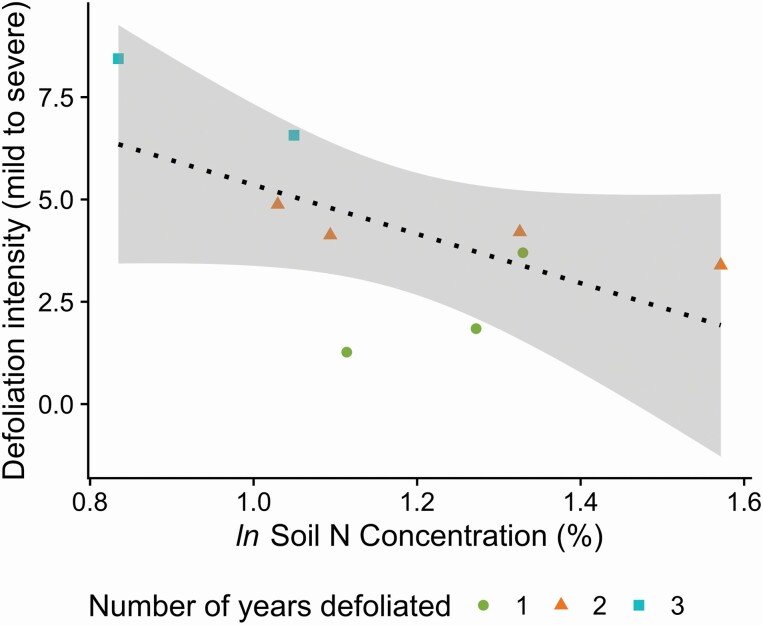
Cumulative defoliation intensity from 2015 to 2018 at the plot scale (*n* = 9 plots) was negatively correlated with soil % N (*r* = −0.65, *P* = 0.060). This pattern suggested that there was a similar inverse relationship between defoliation and soil N at the plot level that was also found at the tree level (Fig. 2).

At the spatial scale of forest plots, there was a significant negative correlation between soil N concentration and the cumulative defoliation intensity from 2015 to 2018 (Fig. 3, *r* = −0.65, *P* = 0.060). This trend paralleled the negative relationship between defoliation and soil N concentrations associated with individual trees (Fig. 2B). There was not a significant relationship between the soil N concentrations and the number of sequential years of plot-level defoliation (Fig. 3, *P* = 0.18, *r*^2^ = 0.25).

**Figure 4.**
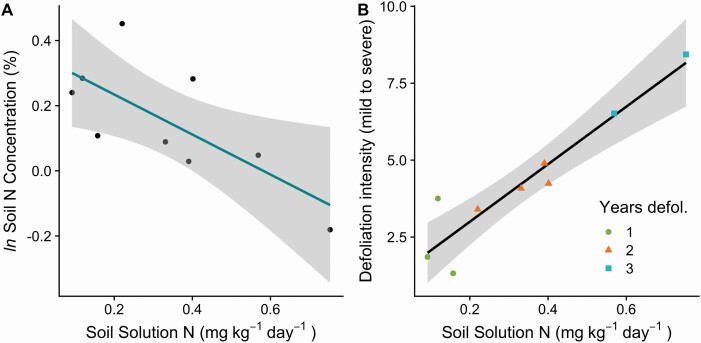
(A) There was a significant negative relationship between plot-level soil N concentrations and the amount of N in soil solution that accumulated between early June and late August (*r* = −0.73, *P* = 0.026, *n* = 9 plots). (B) There was a significant positive relationship between N in soil solution, the defoliation intensity and the number of years defoliated (*r* = 0.94, *P* < 0.01, *n* = 9 plots).

Plot-level soil solution N availability was significantly negatively correlated with the plot-level soil N concentrations (Fig. 4A, *r* = −0.73, *P* = 0.026), and soil solution N availability was significantly positively correlated with plot-level cumulative defoliation intensity (Fig. 4B, *r* = 0.94, *P* < 0.01). We found a strong positive effect of the number of sequential years of defoliation on soil solution N availability, where plots that were defoliated for three sequential years had amounts of inorganic N in soil solution that were up to 400 % larger than plots that were defoliated for only 1 year (Fig. 4B, *F*(2,6) = 26.2, *P* < 0.01, *r*^2^ = 0.86).

## Discussion

### Nitrogen concentrations and defoliation severity

Overall, there was a consistent negative correlation between soil N concentrations and defoliation severity at the tree and plot scales, where trees and plots with higher soil N concentrations were associated with less intense defoliation (Figs 2B and 3). This result was in contrast to our hypothesis that higher ecosystem N concentrations would be associated with higher rates of caterpillar herbivory. While results from laboratory studies testing caterpillar herbivory preference with respect to leaf N concentrations have been mixed (Lindroth *et al.* 1997; Foss and Rieske 2003; Milanović *et al.* 2014), our results suggest that trees and plots with lower soil N concentrations experienced more herbivory and overall defoliation. A potential mechanism for the observed pattern is that instead of travelling in search of higher N quality leaves, the caterpillars consume the leaves that they have access to and consume a relatively larger quantity of leaf mass to meet their nutritional requirements. This interpretation is in line with research that indicates that although *L. dispar* caterpillars travel within a tree, there is very little caterpillar dispersal between trees aside from initial wind dispersal, which is not directed dispersal and therefore does not select for high-quality trees (Weseloh 1987).

Though we found evidence of a relationship between soil N concentrations and *L. dispar* defoliation, it is challenging to infer causality since local soil N concentrations can change in response to tree defoliation. A prior field experiment on *Quercus* defoliation effects found that inorganic and soil N concentrations initially declined in the months following defoliation, potentially due to tree-regulated changes in root growth, activity, turnover or rhizodeposition in response to herbivory (Frost and Hunter 2004). However, in the subsequent year following the experimental defoliation, the soil N reservoir at 0–5 cm depth was a large sink for enhanced N deposition from insect frass (Frost and Hunter 2007).

Although the soil N concentrations were negatively associated with defoliation, the leaf total N concentrations were not significantly correlated to herbivory intensity (Fig. 2A). This pattern is potentially due to the lower range of variation in the leaf total N concentrations compared to the soil total N concentrations, and/or factors of leaf location (Niinemets 1997), seasonality (Xu and Griffin 2006; Yang *et al.* 2017), and/or phytochemical responses (Frost and Hunter 2008; War *et al.* 2018) that could have influenced our measurements of leaf N concentrations, or low sample size. Previous studies of forest N cycling using δ ^15^N have suggested that the below-ground soil N dynamics are a better indicator for ecosystem N cycling compared to above-ground N dynamics due to integrative lags in soil processes (Templer *et al.* 2007). In addition to the sources of measurement variation addressed in the previous section, this would also potentially help to explain the lack of coherence between soil and leaf N concentrations and the lack of a significant relationship between leaf N concentrations and herbivory in comparison to soil total N concentrations and defoliation (Fig. 2A and B).

Landscape-level studies have found that N levels in leaves are positively correlated with rates of atmospheric N deposition (Pitcairn *et al.* 2001), and that higher leaf N concentrations caused by the addition of N from human activities can lead to an increase in the size of insect populations (Throop and Lerdau 2004). However, results from this study are more in line with leaf-level studies that indicate that feeding rates of caterpillars increase when leaf N quality is lower (Lindroth *et al.* 1997). Recent research has found decreasing rates of atmospheric N deposition in North America (Lloret and Valiela 2016) and lower N availability in Northeastern forests (Durán *et al.* 2016; Elmore *et al.* 2016), which could potentially further increase defoliation intensity.

### Plot soil solution nitrogen availability and defoliation severity

Soil solution N reflects the availability of inorganic N to plant roots, whereas the soil total N concentration represents the long-term pools of N in the soil that are mostly composed of soil organic matter. Defoliation transfers N from the canopy to the soil, particularly in the forms of frass (caterpillar faeces), green leaves that fall, and insect biomass. An increase in availability of soil N for trees with defoliation could potentially help trees to recover following stress from herbivory. Within forest plots, cumulative defoliation intensity and soil solution N availability were significantly positively correlated (Fig. 4B). There was also a strong relationship between the number of years of sequential defoliation and soil solution N availability, where plots that were defoliated 3 years in a row had much higher amounts of soil solution N than plots defoliated 1 or 2 years.

Other studies have found ephemeral enhancements in inorganic N available in soil solution in the months following *L. dispar* defoliation (Lovett *et al.* 2002; Russell *et al.* 2004). In both studies, frass N was quickly taken up from the soil with enhanced plant N uptake in a poplar plantation (Russell *et al.* 2004) and elevated microbial N uptake and immobilization in a mixed hardwood forest (Lovett *et al.* 2002; Christenson *et al.* 2002). Both studies found that the N redistributed to the soil solution inorganic N pool by a single severe defoliation event was not lost by leaching at the ecosystem and watershed scales (Lovett *et al.* 2002; Russell *et al.* 2004). A legacy of elevated inorganic N was also observed in birch forests that experienced 2–3 severe sequential defoliation events and was explained as enhanced decomposer activity (Kaukonen *et al.* 2013). The pattern in our study could potentially be the result of lag effects following defoliation where N mineralization might be enhanced through higher mortality in tree fine roots or more dynamic microbial growth and mortality in microbial population cycles.

There could be alternate explanations for the positive relationship between soil solution N availability and defoliation severity found in this study, such as other site conditions that influence available N. For example, it is possible that particular site conditions such as soil moisture influence soil N cycling (Landesman and Dighton 2010) and that these conditions also co-vary with favourable conditions for *L. dispar* growth and survival at these plots. It is also possible that the severe tree stress caused by 3 years of sequential defoliation led to higher rates of root mortality, which could represent an additional pulse of organic matter to the soil N pool and the higher availability of soil solution N observed in our study. The results from our study highlight the utility of field observations and experiments that capture high and recurrent disturbance severities and monitor changes in ecosystem processes over several years.

### Coherence with ecosystem N cycling at trees and plots

In comparing leaf-level to tree-level caterpillar defoliation measurements, we found that individual leaf-level caterpillar herbivory was significantly positively correlated with assessments of tree-level defoliation (Fig. 2D). This pattern suggested that the impact of caterpillar herbivory of individual leaves scales proportionally to overall defoliation at the tree level, even when leaves were only collected from branches that could be reached from the ground.

We found no correlation between soil and leaf total N concentration at the scale of individual trees (Fig. 2C). Our measurements for the leaf total N concentrations could be impacted by the collection of leaves that could only be reached from the ground, since shade leaves tend to have lower N concentrations than sunlit leaves (Niinemets 1997). Trees that experience herbivory have also been observed to reduce leaf N concentration in response to defoliation (Frost and Hunter 2008) and phytochemical induction of plant defences can also impact leaf N concentrations (War *et al.* 2018). However, another study found that hemlock foliar N increased following herbivory by hemlock woolly adelgid (Schaeffer *et al.* 2017). Additionally, leaves in this study were collected at the end of the growing season in October 2018, when leaf N concentrations are typically at the lowest levels of the season, particularly compared to the levels in May and June when L. dispar larvae were feeding (Scriber and Slansky 1981; Xu and Griffin 2006; Yang *et al.* 2017). We found that the values of *Q. rubra* foliar N concentrations (mean = 1.57, SD = 0.255) in this study were about 30 % lower than *Q. rubra* foliar N measurements from a prior Harvard Forest study conducted from 1988 to 1991 (mean = 2.28, SD = 0.23; Martin and Aber 1992). Together, the factors of shaded leaf collection, the seasonal timing of collection during autumn and/or phytochemical responses to defoliation could help to explain the lower-than-expected concentrations of leaf N and lack of a relationship with defoliation.

Across the different forest plots, soil total N concentrations and the amount of N in soil solution were negatively correlated (Fig. 4A). This pattern could be due to the fact that the soil organic matter contains most of the total soil N, and a primary source of inorganic N in soil solution is the decomposition of this soil organic matter (McClaugherty *et al.* 1985; Aber *et al.* 1990). Therefore, it is consistent that forest plots with higher amounts of soil solution N have lower soil N concentrations, while soils with larger N concentrations have lower amounts of N in soil solution. The measurements of ammonium and nitrate in soil solution in this study were within the same range of variation as other studies that used this same method in mixed Northeastern US forests (Sanders-DeMott *et al.* 2018).

### Ecosystem level implications

*Lymantria dispar* defoliation events have both short- and long-term impacts on forest dynamics. In the short term, defoliation events can alter canopy light transmittance, temperature and moisture content of the soil, and forest nutrient cycling (Gandhi and Herms 2010). Over time, *L. dispar* defoliation events in the Northeast can lead to large-scale oak mortality and changes in forest stand composition and dynamics including a decrease in oak dominance (Barron and Patterson 2008; Morin and Liebhold 2016). Changes in forest composition due to oak mortality from *L. dispar* outbreaks are predicted to negatively impact eastern North American wildlife, which rely heavily on the consumption of acorns from oak masts (McShea *et al.* 2007). Increased climate variability including drought events may impact the intensity and frequency of *L. dispar* outbreaks because one of the main controls on *L. dispar* populations, the fungal pathogen *Entomophaga maimaiga*, proliferates with higher levels of precipitation (Hajek and Humber 1997; Reilly *et al.* 2014).

Variation in defoliation rates across the broader region of Northeastern US forests could drive variation in relationships between defoliation and ecosystem N cycling (Davidson *et al.* 2001). Patterns within our study could also have been influenced by trends in regional patterns of declining N deposition in Northeastern US forests (Lloret and Valiela 2016) where impacts of climate change, including increasing temperatures, lower snow accumulation and changing seasonality, may be contributing to a decline in N availability (Durán *et al.* 2016; Elmore *et al.* 2016). This pattern has implications for our study because of the potential relationship between higher rates of defoliation at lower soil N concentrations, which could predict a larger extent of defoliation with future declines in N pollution.

## Conclusions

Overall, we found that at the scales of individual trees and forest stands, there was a consistent negative relationship between soil N concentrations and defoliation intensity. We found no significant relationship between leaf N concentrations and the amount of herbivory. This study provides important *in situ* characterization of nitrogen cycling at multiple scales that could help to improve predictions of nutrient cycling within ecosystem models in response to recurrent and severe landscape-level defoliation events. Declines in dominant forest canopy species like oaks can have widespread impacts on forest ecosystem processes such as decomposition, carbon sequestration and nutrient cycling, as well as on processes in nearby aquatic ecosystems (Ellison *et al.* 2005). Forest insects and pathogens also impact ecosystem services such as water quality and carbon sequestration (Dale *et al.* 2001), which are highly influenced by ecosystem N cycling. Additionally, changes in climate can directly impact the *E. maimaiga* fungal control to *L. dispar* populations, potentially increasing the likelihood of future *L. dispar* outbreaks (Hajek and Humber 1997; Reilly *et al.* 2014). The strong relationships we found between ecosystem N cycling and defoliation following a severe, multi-year invasive insect irruption highlight the importance of investigating the long-term effects of severe and recurring ecosystem disturbances. Particularly considering that these types of disturbances are likely to increase in frequency and intensity, long-term monitoring efforts are critical to understanding ecosystem feedbacks and potential thresholds.

## Data Availability

Data associated with this manuscript are available through the Environmental Data Initiative (Conrad-Rooney *et al.* 2020a) and in the Harvard Forest Data Archive (Conrad-Rooney *et al.* 2020b).

## Notes

**Plants, Ecosystems & Climate. Chief Editor:** Mary Heskel
